# Risky Sexual Behaviors among Female Youth in Tiss Abay, a Semi-Urban Area of the Amhara Region, Ethiopia

**DOI:** 10.1371/journal.pone.0119050

**Published:** 2015-03-04

**Authors:** Gojjam Tadesse, Bereket Yakob

**Affiliations:** 1 Addis Continental Institute of Public Health, Bahir Dar, Ethiopia; 2 School of Nursing & Public Health, Howard College, University of KwaZulu-Natal, Durban, South Africa; 3 Health Economics and AIDS Research Division (HEARD), University of KwaZulu-Natal, Durban, South Africa; International AIDS Vaccine Initiative, UNITED STATES

## Abstract

**Background:**

Little is known about sexual risks and associated factors about female youths in semi-urban areas of Ethiopia. This study aimed to describe the nature and magnitude of risky sexual behaviors, and the socio-demographic and behavioral determinants among female youths in Tiss Abay, a semi-urban area on the outskirts of Bahir Dar City of the Amhara Region in northern Ethiopia.

**Methods:**

A cross-sectional census type study was conducted among female youths who were unmarried and aged 15–29 years in September 2011.

**Results:**

711 female youths participated in the study, with the mean age of initiation of sex of 78.6% being16.73±2.53 years. Only 52(9.3%) used condom during the first sex. Within the last 12 months, 509(71.6%) had sexual intercourse and 278(54.6%) had two or more sex partners, and 316(62.1%) did not use condom during their last sex. Sex under the influence of substances was reported by 350(68.8%), and a third of the recent sexes were against the will of participants. One or more risky sexual practices were reported by 503(70.3%) participants, including: multiple sexual partnerships, inconsistently using or not using condoms, sex under the influence of alcohol and/or sex immediately after watching pornography. Age group, current marital status, drinking homemade alcohol, chewing ‘khat’, watching pornography and using any form of stimulant substances were the predictors of risky sexual behavior. Watching pornography before sex and sex for transaction were the predicators of not using condom during most recent sex.

**Conclusions:**

Risky sexual behaviors were very common among the female youths in Tiss Abay. Initiation of context-based interventions, such as raising awareness about the risks, safer sex practices, condom promotion and integration of gender issues in the programs are recommended.

## Introduction

The youth population constitutes approximately one third of the world population and is a productive force for the world economy[1]. The absence of a universally accepted definition of ‘youth’ requires comparisons among studies to be handled with caution, those that are available being determined by the purpose of the project in which they are used[2–4]. For the purposes of this study in Ethiopia, as defined by the Ministry of Youth and Sports, they were identified as those from the ages of 15–29.

Risky sexual behavior is defined as an individual’s conduct that increases the susceptibility of the person to sexually transmitted infections (STIs) and HIV, unwanted pregnancy and psychological distress[5–10]. According to published research, risky sexual behaviors may present as having unprotected sex (without or inconsistently using a condom), having multiple sexual partners, having sex under the influence of stimulant substances, or having sex immediately after watching pornographic media[5–10].

Evidences show that youth are most likely to engage on risky sexual behaviors when compared to adults[5]. The earlier the initiation of sex, the more risky the behavior is likely to be and the greater the chance of acquiring sexually transmitted infections[9]. In the developing world, the risks of HIV/STIs to youth are higher due to poverty, lack of education, gender inequality and resource scarcities to promote safer sex practices[11–14].

Risky sex behavior may result from being easily influenced by peers, poor bonding with and limited support from parents, inappropriate parenting roles and role models, and living in unfavorable environments[7,8,10,15–19]. Those who perform poorly at school, are from disadvantaged communities and seek attention from peers and men are more likely to engage on risky sexual activities[15]. Due to lack of awareness about the risks associated with unprotected sex, youth often acquire sexually transmitted infections, while the young girls may have unwanted pregnancies[5,6]. This behavior may also be due to a lack of adequate information and basic skills to deal with their emotions, and high peer pressure to experiment with sex[1,7,8,20]. Social norms in which the youth live and grow can heighten the risks even if youth have sufficient education[21].

Female youths bear disproportionately heavy social, economic and psychological burdens, specifically in developing countries due to poor emphasis given to their education and prevailing violence against them[5,10–14]. This can be exacerbated in rural areas, where there is extremely less chance of getting an education, there are impoverished living conditions, and services and infrastructure are limited or absent[7,10,11]. In these circumstances, a lack of awareness about STI risks, the inaccessibility of condoms, a lack of skills to address pressures from peers and men[8,11,22], unfair treatment from family (not sending to school, assigning all home duties to young girls, not providing emotional support, etc.), and sexual abuse of women[5,11,23] may be common, facilitating risky behavior.

The risk of acquiring STIs can be substantially reduced by using condoms correctly and consistently during all sexual intercourses. In this regard, while global, regional and national efforts to distribute and make condoms available have increased in recent years, youth in developing countries still face considerable challenges to access and use condoms[24]. Due to the conservative social values associated with private matters that result in taboos regarding public talks about sex and condom, the youth in developing countries are not exposed to the social platforms and opportunities to hear about and engage with discussion about sex and condoms. These attitudes result in condoms not being available in public places and shops, and when they do have access to them, they lack the knowledge and skills about how and when to use them.

Problems related to condom use may be exacerbated when youths consume substances that reduce their inhibition and affect their self-control such as ‘khat’ leaves (Catha Edulis—a green plant that grows in Yemen and East Africa)[25,26], alcohol, marijuana and others[27–30]. These substances can affect decision making before and during sex that impacts on condom use and exposes them to risky sexual acts. Studies in Ethiopia show that the odds of engaging in risky sexual behavior was 3.4 times higher among alcohol consumers[29] and 2.3 times among ‘khat’ chewers[28] compared to non-consumers of these substances.

Condom use may also be highly influenced by the type and intensity of the relationships being felt and experienced by partners[19]. Apart from individual beliefs, the respect shown for each person’s values within the relationship plays a substantial role about the use of condom[19,31]. Some female youths may agree to not use condoms based on the belief that their partner may not like it. They may also try to preserve the relationship at the expense of condom use[19,31,32], resulting in their health being sacrificed. As the body of literature indicates, partner dynamics such as the type of partner, complexities of condom negotiation with respect to risk and trust, power dynamics and the type of the relationship present challenges to the youth and increase the risk of acquiring STIs and having unwanted pregnancy[19,31,32].

Furthermore, traditional values and socio-cultural expectations can result in women, especially young women, being considered and treated as inferior, resulting in sexual risk taking, and being the victims of violence and sexual harassments in sub-Saharan Africa[10–14,33]. In such conditions, women are supposed to show their decency by remaining virgin and not being sexually active until marriage[33,34]. Even after marriage, in most cases, sexual intercourse is at the discretion of the men, without entertaining women’s values or desires. On the contrary, men are expected to be masculine and sexually active at all times[22,35]. Thus, male youth or men may begin demonstrating and fulfilling such expectations and roles by tempting, bullying and/or raping female youths at very young age, which contradicts the idea of women remain virgin until marriage.

Young women in Africa may also have sex with various people, including their marital and causal partners, and friends for various reasons, including curiosity, resource or financial transactions, pressure[8,22,35] and violence[33]. When female youths do not know how to protect themselves, and when they are not empowered and are forced to have sex against their will, the sex will be riskier[35,36].

Research reports showed 3.4% and 1.4% HIV prevalence rates among women and men, respectively in sub-Saharan Africa in 2009[36]. In 2011, the Ethiopian Demographic and Health Survey (EDHS) showed that women were disproportionately affected by HIV i.e. adult HIV prevalence was 1.9% among women while only 1.0% among men. The same study also indicated that having multiple sex partners was highest among women aged 15–29 years, and that 53.0% of women who had multiple sexual partners in the past 12 months did not use condom[37].

Studies in Ethiopia regarding risky sexual behavior have been concentrated in urban centers and school environments. Less is known about sexual risks and associated factors among female youths in semi-urban areas of Ethiopia. The aim of this research was to describe the nature and magnitude of risky sexual behaviors, and the socio-demographic and behavioral determinants among female youths in Tiss Abay. This is a semi-urban area on the outskirts of Bahir Dar City of the Amhara Region in northern Ethiopia.

## Methods

### Study Area

This study was conducted in Tiss Abay Town, which is one of the satellite town of Bahir Dar City Administration, and is located 30 km southeast of Bahir Dar City, Amhara Region, northern Ethiopia. The town is a small emerging urban setting, which may be considered to be rural area as it has limited infrastructures and poor living conditions. The town is divided into A, B and C blocs for administrative purpose, and according to the 2010 population projection based on the 2011 population and housing census, there were 2,981 households with 13,414 inhabitants, of whom 7,144 were males and 6,270 were females in 2011. Approximately 2,074 households were male headed and 907 were female headed, with most livelihoods being based on subsistence farming and small scale businesses.

### Study Design and Participants

A quantitative, cross-sectional, using a questionnaire based survey was conducted in September 2011 on female youths aged 15 to 29 years who were unmarried (including widowed, divorced and separated). Married females were excluded from the study as they had different sexual behavior and related risks. Only female youths who were permanent residents, who lived continuously in Tiss Abay at least for six months prior to the study time were included. Temporary residents were excluded to avoid the influence of experiences, knowledge and practices from outside the study area.

### Sampling

In this household survey, all (740) female youths who met the above criteria were identified and invited to participate. This sample size was deemed adequate, as it exceeded the required(calculated) sample size of 641 with the assumption of an 80% power of study, 95% confidence interval, 4% margin of error, α-error of 0.05. It also accounted for the 58.5% prevalence of not using or inconsistently using condoms among out-of-school youth who had sex with causal partner in Bahir Dar City in 2004[1]. A 10% allocation was made for non-responses to the results computed using single proportion population formula for sample size determination in cross-sectional studies.

### Data Collection Instruments

An anonymous, structured, interview questionnaire was developed after reviewing relevant literatures that reviewed key variables as well as earlier studies on risky sexual behaviors among youth[1,5,7,8,18,19,22]. The questionnaire was grouped into six categories according to the study objectives. The six categories consisted of: demographic characteristics (12 questions); sexual history and behavior (16 questions); knowledge and attitudes about HIV (six questions); condom utilization (six questions); knowledge about other sexually transmitted infections (three questions); and substance use (19 questions).

The questionnaire contained Yes/No/I don’t know answers options, multiple response questions and opinion questions. For opinion and attitude questions, balanced Likert scales were used to ensure participants respond freely. The questionnaire was translated into Amharic (which is a local and national language) and back translated into English to check for accuracy of the translation. The questionnaire was pretested in Meshenty Town (another satellite town of Bahir Dar City), which had similar characteristics to Tiss Abay in terms of the variables of interest, after which minor modifications were made.

### Data Collection

Nine female data collectors who were fluent in the local language and familiar with the culture, and had completed Grade 10 schooling were identified, hired and trained. Female data collectors were preferred to males due to the sensitive nature of the questions and unfavorable local contexts regarding a man interviewing a woman about sex related issues. Two supervisors who had completed a college education were hired from Tiss Abay to supervise data collection and provide support for the data collectors. The principal investigator (PI) coordinated the data collection, made site visits, and oversaw the whole process. Data collectors and supervisors were trained for three days on the research ethics, the study objectives, data collection principles and processes, as well as the research instruments.

### Data Analysis and Interpretation

The data were checked and edited, then coded, entered into SPSS, cleaned and checked for outliers and completeness, after which were analyzed using SPSS Version 16 (SPSS Inc., Chicago, IL, USA). The frequencies of different variables were determined, the crude and adjusted odds ratios, and 95% confidence interval were computed to determine any association between the variables. Bivariate analysis to measure the strengths of associations, and multivariate analysis to adjust for the confounders were conducted. X^2^(chi-square) test was used to identify the statistical significance of the associations between the variables. During the multivariate analysis, for all logistic regression analysis, a ‘conditional forward’ model was used. The cut-off point used for statistical significance was p< 0.05.

### Ethics Statement

Before the study was initiated, ethical clearance was obtained from the Ethical Committee for Research of the Addis Continental Institute of Public Health, Ethiopia. A permission letter to conduct the study in the town was also obtained from Bahir Dar City Administration, and was presented to all participants and stakeholders by the data collectors and the principal investigator as needed.

Interviews were conducted face-to-face in private at the identified houses after outlining the study objectives and reading the information sheet. Verbal consent was obtained from all uneducated participants, an educated adult from the family or a community health worker acted as a witness, with the data collector ticking a checkbox as a sign of agreement when the participant consented. For all educated participants, written consent was obtained. For the participants who were under 18 years, consent was obtained from the parents or guardians. Completed consent forms were detached from the questionnaire, a copy being given to the participant, and kept in a locked cabinet on completion of the interview. The consenting process was reviewed and approved by the Committee for Research Ethics. The softcopies of the database were kept in a password protected computer.

## Results

The results are presented with respect to the participants socio-demographic characteristics, initiation of sex and recent sexual behavior, substance use and sexual behaviors, factors associated with risky sexual behavior, and condom use and associated factors.

### Socio-demographic Characteristics

Of the 741 female youths identified as eligible to participate, 711(95.9%) gave consent and participated. Their mean age was 21.54±3.84 year, approximately 37.6% did not attend any formal education, 80.2% were Orthodox Christians and 43.6% were living alone at the time of the study. Details of the socio-demographic characteristics are presented on Table 1.

**Table 1:** Socio-demographic characteristics of the female youth.

*Socio-demographic Characteristics*	*Frequency (%)*
*Age Group (n = 711)*	
15–19 years	228(32.1)
20–24 years	300(42.2)
25–29 years	183(25.7)
Mean = 21.54±3.84	
*Migration from other areas (n = 711)*	
Yes	362(50.9)
No	349(49.1)
*Length of stay in Tiss Abay (n = 711)*	
≤ One Year	139(19.5)
≥ One Year	572(80.5)
*Education (n = 711)*	
No formal education	267(37.6)
Primary education (Grade 1–8)	241(33.9)
High school education	139(19.5)
University/College/Vocational	64(9.0)
*Occupation (n = 711)*	
Self-employed*	222(31.2)
Paid Stable Employment	48(6.8)
Daily Laborer	140(19.7)
Student	215(30.2)
Unemployed	86(12.1)
*Religion (n = 711)*	
Orthodox Christian	570(80.2)
Muslim	125(17.6)
Protestant Christian	16(2.3)
*Father Educated (n = 711)*	
Yes	317(44.6)
No	394(55.4)
*Own income sufficiency^**∞**^ (n = 711)*	
No	471(66.2)
Yes	240(33.8)
*Currently living (n = 711)*	
Alone	310(43.6)
With relatives	119(16.7)
With parents	219(30.8)
With sexual partner	36(5.1)
With friend/s	27(3.8)

* own farm & small businesses;

∞ perceived income sufficiency for basic needs i.e. food, accommodation and schooling.

### Initiation of Sex and Recent Sexual Behavior

Of the 711 participants, 559(78.6%) had initiated sex, the age at initiation ranging from 10–28 years, with the mean age at which this had occurred being 16.73±2.53 years. The frequency distribution revealed that 46.5% of the sexual initiations occurred from age 14–16 years, and that approximately 94.6% the sexual initiations happened by or at the age of 20. Among those who initiated sex, only 52(9.3%) had used condoms, while 497(88.9%) and 10(1.8%) reported that they did not use and did not remember using condom respectively during their first sexual intercourse. Regarding the single major reason for the first sex act, the responses were as follows: curiosity 141 (25.2%); marriage 202(36.1%); peer pressure 111(19.9%); alcohol/substances influence 8(1.4%); coercion by men 25(4.5%); and a transaction (in form of money or kind) from men) 72(12.9%).

Recent (in the last 12 months) sexual behavior indicated that 509(71.6%) had had sexual intercourse during that time. When asked about the type of recent sex partner/s, 299(58.7%) reported casual and 377(74.1%) indicated regular partners. A total of 168(33.0%) participants reported having concurrent or mixed sexual partners (sex with both causal and regular partner) in the last 12 months. Regarding the number of recent sex partners, 231(45.4%) had one partner, while 278(54.6%) had two or more partners. Only 193(37.9%) reported using condoms in their most recent sexual encounter, while 316(62.1%) had not used condoms.

Of those who used condoms, only 70(36.3%) used them consistently. The leading reasons (multiple responses) for recent sex were peer pressure (270, 53.0%), followed by transaction (money or material) (242, 47.5%) and coercion 173(34.0%). Multiple responses were reported by participants for not using condom during the recent sex, and included “didn’t like condom” (75.6%), a dislike for condoms by their partners (40.5%) couldn’t access condom (15.8%) and not having money to buy them (7.8%).

### Substance Use and Sexual Behaviors

Of the 711 participants, 350(68.8%) had sex in the last 12 months under the influence of substances such as alcohol, khat or cigarettes. Homemade alcohols (Tella, Tej’ and ‘Areke’) were used by 383 (53.9%) participants, and alcohol use was approximately 61.6%. Substance use (alcohol, ‘khat’ and smoking) prevalence among the participants was 66.0%(469), with the details being presented in Table 2.

**Table 2:** Substances used by the female youth.

Types of substances used in the past 12 months	Frequency (%)
Homemade alcohol*	383(53.9)
Beer (bottled)	148(33.9)
Any form of alcohol	438(61.6)
Khat (chewing)	171(24.1)
Smoking Cigar	66(9.3)
Substance use (any of the above)	469(66.0)

* Homemade alcohol included ‘tej’, ‘tella’ and ‘areke’—these drinks have extremely high alcohol content compared against beer.

Recent (in the past 12 months) risky sexual behaviors among female youths in Tiss Abay are presented on Table 3. Not using or inconsistently using condoms was reported by 316(62.1%) of the participants, with 278(54.6%) having multiple sexual partnerships (two or more partners). Substance use, 342 (99.8%); watching pornographies, 222(73.3%); and sex without using a condom for extra money and/or gift, 100(42.0%) were reported by participants as shown on Table 3. One or more risky sexual behaviors were reported by 503(70.7%) of the respondents, accounting for 98.8% of those who had sex in the last 12 months.

**Table 3:** Risky sexual behavior in the last 12 months among the female youth.

Risky sexual behavior	Frequency (%)
Multiple sex partners (n = 509)	278(54.6)
Not using condom in the most recent sex (n = 509)	316(62.1)
Inconsistently using condom (n = 509)	123(24.2)
Not using or inconsistently using condom (509)	439(86.2)
Sex immediately after drinking alcohol (n = 397)	310(78.1)
Sex immediately after chewing ‘Khat’ (n = 165)	138(83.6)
Sex immediately after smoking (n = 65)	50(76.9)
Sex without condom for extra money or gift (n = 238)	100(42.0)
Sex immediately after watching pornography (n = 303)	222(73.3)
Not using condom after chewing ‘Khat’ (n = 165)	77(46.7)
Sex after consuming the above substances (n = 344)	342(99.4)
Not using condom after watching pornography (n = 303)	113(37.3)
Had at least one risky sexual behavior*	503(98.8)

* Multiple sexual partner, sex under the influence of substance, not using or inconsistently using condom during sex, sex without condom for extra money or gift and sex after watching pornographies.

### Factors Associated with Risky Sexual Behavior

Bivariate and multivariate analysis were conducted to examine the relationships between risky sexual behaviors and the socio-demographic variables, and to control for confounding variable respectively. Educational status, religious affiliation, duration of stay in Tiss Abay, parents education, perceived own and family income, perceived sufficiency of income for basic needs, and living arrangements were not statistically associated with risky sexual behavior among the study participants.

In the bivariate analysis, age group, current marital status, employment status, migration status, drinking homemade alcohol, ‘khat’ chewing, watching pornographies and using any form of stimulating substances showed very strong associations (p<0.001) with risky sexual behavior. After conducting multivariate analysis, age group, current marital status, drinking homemade alcohol, chewing ‘khat’, watching pornographies and using any form of stimulant substances remained the predictors of risky sexual behavior among female youths in Tiss Abay. Employment and migration status appeared as confounders (Table 4).

**Table 4:** Multivariate analysis of associated factors for risky sexual behavior.

Variables	Risky Sexual Behavior		Crude OR (95% CI)	Adjusted OR (95% CI)
Variables	Yes (n)	No (n)		
*Age Group*				
15–19 years	83	145	1.00	1.00
20–24 years	258	42	10.73[7.03–16.39]***	3.13[1.76–5.61]***
25–29 years	162	21	13.48[7.94–22.86]***	3.48[1.67–7.25]***
*Current Marital Status*				
Single	193	157	1.00	1.00
Divorced/Separated	191	34	4.57[2.99–6.96]***	3.01[1.62–5.60]
Widowed	119	17	5.69[3.28–9.87]***	2.55[1.20–5.39]*
*Employment Status*				
Self-employed	178	44	1.31[0.72–2.63]	
Paid Stable Employment	43	5	2.78[0.97–7.93]	
Daily Laborer	129	11	3.79[1.72–8.33]***	
Student	88	127	0.22[0.12–0.39]***	
Unemployed	65	21	1.00	
*Migrated from other places*				
No	212	137	1.00	
Yes	291	71	2.65[1.89–3.71]***	
*Drink Homemade Alcohol*				
No	159	169	1.00	1.00
Yes	344	39	9.37[6.31–13.93]***	6.31[3.63–10.99]***
*Chewing Khat*				
No	339	201	1.00	1.00
Yes	164	7	13.89[6.39–30.19]***	3.56[1.39–8.85]**
*Watching Pornographies*				
No	200	178	1.00	1.00
Yes	303	30	8.99[5.87–13.76]***	7.63[4.26–13.66]***
*Current Substance Use*				
No	85	157	1.00	1.00
Yes	418	51	15.13[10.22–22.42]***	4.98[2.94–8.42]***

* p<0.05.

** p<0.01.

*** p<0.001.

### Condom Use and Associated Factors

The bivariate and multivariate analyses were conducted to identify factors associated with current condom use. Age group, education, own and family income, religion, employment and education status were not associated with current condom use. However, current marital status, number of sexual partners (last 12 months), current substance use (alcohol, smoking or chewing ‘khat’), sex for transactions (money or kind) and watching pornographies before sex were significantly associated with condom use during bivariate analysis. Only watching pornography before sex and transactional sex remained predicators of condom use in recent sex after adjusting for confounders by using conditional forwarding model during multivariate analysis (Table 5).

**Table 5:** Multivariate analysis of factors associated with condom use during most recent sex.

Variables	Condom Use		Crude OR (95% CI)	Adjusted OR (95% CI)
Variables	Yes (n)	No (n)		
*Current marital status*				
Single	89	106	1.00	
Ever married^**ɫ**^	104	210	0.59[0.41–0.85]**	
*Watching pornographies* before sex				
No	109	106	1.00	1.00
Yes	22	210	0.39[0.22–0.67]***	0.39[0.22–0.68]***
*Current substance use*				
No	170	252	1.84[1.10–3.08]*	
Yes	23	63	1.00	
*Number of sexual partners*				
One	75	156	1.00	
Two or more	118	160	1.53[1.07–2.21]*	
*Sex for money or kind*				
No	61	158	1.00	1.00
Yes	132	158	0.46[0.32–0.67]***	0.39[0.26–0.58]*

* p<0.05.

** p<0.01.

*** p<0.001.

^ɫ^ divorced, separated or widowed.

## Discussion

The data are discussed with respect to the same order as the Results, namely initiation of sex and recent sexual behavior, substance use and sexual behaviors, factors associated with risky sexual behavior, and condom use and associated factors.

### Initiation of Sex and Recent Sexual Behavior

The majority of participants were sexually experienced during the study period, with sexual initiation starting at early age. This high percentage of early sexual initiation during their early teenage may indicate that sex was carried out with little regard for what can happen and its outcomes and in unfavorable environments. Early initiation of sex and not using condom can be very risky and the youth may get STIs/HIV and/or unwanted pregnancy in their first sex at very young age[5,6,9,23,31]. The study findings suggest that sex education (including what risky sex is and how to make it safer) needs to be initiated in early adolescence, continued throughout the youth age to reduce sexual risks and vulnerability to STIs/HIV.

Similar to reports from others areas in Ethiopia and abroad[1,8–10,16,19], female youths in this study initiated sex for reasons such as curiosity, peer pressure and transaction. These factors could be related to lack of awareness of safer sex and condom use, emotional and economic problems, power imbalances and gender roles, and inter-generational relationships[7,8,11,13,14]. Being from disadvantaged community and where there were operating gender and social class differentials (age, education status, income, gender power, etc.), the youth might be depending on their sexual partners’ interests and taking sexual risks[38]. Interventions focusing on empowering the youth, including trainings on assertive communication and negotiation skills, self-efficacy and life skills are advised to increasing coping during pressures and bargaining power in sex[38,39].

One third of study participants had their first sex when they got married, which may indicate that the traditional values of virginity[34] or ‘abstinence’ can still be considered as a healthy choice for some youths in semi-urban settings in Ethiopia. Peer pressure and transaction being the main reasons for the most recent sex may indicate the importance of peer-based interventions in promoting healthy sexual behavior[8,9].

Similarly, one third of participants reported that they recently had sex against their will, which indicates the presence of violence against women (sexual harassment, rape and else), highlighting the power dynamics and status of women in poor socio-economic communities. It also raises the issue of the traditional social justification of forced sex, which increases the vulnerability of young women to risky sex[5,10,14,22,33,34,40]. Appropriate legislation, supported by law enforcement infrastructure and community-based interventions may be necessary to address the demeaning attitudes and practices towards women, especially female youths. While empowering women is essential to enable them to make more informed choices, educating men about respectful and responsible sexual behaviors also needs to occur if women’s rights and the consequences of risky behavior are to be addressed. Interventions for women can include education, economic support, legal enforcements and community mobilization[10,11,13,14,34,35,41–44].

Recent (in the past 12 months) sexual behavior of the study participants showed complex patterns of sexual partnerships, number of sexual partners, substance use and condom use. The majority had sex with causal partners, making the sex not well planned and increasing the chance of not using condom. One third of participants had concurrent sexual partners, with reported high proportion of not using condom and multiple partners, increasing the risk of contracting STIs/HIV and the chance of having unplanned and unwanted pregnancy[45–47].

### Substance Use and Sexual Behaviors

The high proportion of female youths who consumed mainly homemade alcohols (‘areke’, ‘tella’ or ‘tej’) which contain very high proportion (up to 45.0%) of alcohol may increase the susceptibility of youth to risky behaviors by affecting their judgments and making them less responsible in sex acts[1,5,21,27–29,48]. Similarly, sex immediately after consuming stimulants (alcohol and ‘khat’), may result not using condom.

Female youth who consumed homemade alcohol were 9.37 times more likely to engage on risky sexual behavior than those who did not (p<0.001). This might be associated with the lack of regulation for homemade brews and their very high alcohol content. In addition, people who drank homemade alcohols might be economically disadvantaged and could not afford better living conditions or standard beers, decreasing their bargaining power in sex and increasing risk taking.

Similarly, the odds of having risky sexual behavior was 13.89 times higher among ‘khat’ chewers than non-chewers (p<0.001). As usual practice in Ethiopia, ‘khat’ chewing is followed by smoking and alcohol consumption[49], with intentions to neutralize its effects (over stimulation of the sensory and motor function, euphoria, hallucinations, lag and difficulty in decision making, lethargy, etc.)[25,26]. ‘Khat’ consumption is also associated with early initiation of sex which in turn is associated with not using condom in Ethiopia[50]. When these substances (‘khat’and alcohol) are combined, they reduce inhibitions and increase the vulnerability of female youth for risky sex[49–51].

The study shows very high susceptibility of substance users to risky sexual behavior as demonstrated elsewhere[16,27–29,48], the odds of having risky sexual behavior was 15.13 times higher among substance users than non-users (p<0.001). The results indicate the importance of integrating substance control programs into reproductive health promotion programs, such as advocacy for substance controls, not selling substances to minors and promoting responsible substance use among older youths.

### Factors Associated with Risky Sexual Behavior

Overall, risky sexual behavior was prevalent among the study participants. The age group 15–19 years was less likely to engage in risky sexual behavior compared to the 20–24 age group and 25–29 year age group. The latter group was more likely to have risky sexual behavior than the first group (p<0.001). The study is unable to provide reasons why the older groups were more likely to engage in risky behaviors. Possible reasons could be increased demand for money, loosening control from parents and increasing independency. Further studies are advised for a better understanding of the association between age and reasons for sexual activity. Our findings suggest that earlier initiation of intervention programs at younger age could help prevent risky sexual behavior among the female youth.

Female youths in the study area who were previously married and divorced or separated were 3.01 times more likely to have risky sexual behavior compared to those who had never married (p<0.01). Similarly, widowed were 2.55 times more likely to engage on risky sexual behavior than single participants (p<0.05). Why these two groups were more likely to have risky sexual behavior may require further studies. It may be that past experiences and complicated partnership dynamics [reasons for divorce/separation], and economic problems could be contributing factors. The results show the importance of promoting safer sex among previously married, separated and widowed youths.

In addition to substance users, the odds of having risky sexual behavior was 8.99 times higher among those who watched pornographies than non-watchers. This suggests that exposure to pornography might affect their decision making regarding sex. In addition, the contents of pornographic media may contain inappropriate and unsafe sexual behaviors i.e. not using condom, sex with causal partner and multiple sexual partnerships that can promote risky behaviors.

Studies elsewhere indicated that some reasons for risky sexual behaviors could be unplanned sex, forced sex, gender-power relationships, lack of good role models, ineffective parenting styles, lack of awareness about risks and low risk perceptions[1,5,8,10,19,22,41]. In this study, when condoms were used, only 36.3% did so consistently, which may indicate a lack of awareness about how and when to use them. Added to the social, economic and attitudinal challenges faced by the youth face, this may fuel their risky sexual behavior.

Similar to other studies[19,31,32,52,53], female youths had various concepts and attitudes about condoms that impact their use during sex. The results of this study show that participants who had sex recently showed unfavorable attitude towards condoms i.e. “don’t like condom”, and indicated that many of their sexual partners felt the same. This high level of unfavorable attitudes may result from the perceptions that condom reduces sexual pleasure, and that using them demonstrates a lack of trust in their sex partner[19,31,32,52,53].

A qualitative study in South Africa indicated possible factors that contribute to these attitudes include gender—power relationships, the role of significant others in affecting the age of initiating sex and with whom, low risk perceptions of unprotected and unplanned sex, peer pressure and low socio-economic status[52]. The problems of condom accessibility and financial resources were also reported in this study as preventing their use. The study findings and these highlight the role that partnership dynamics, such as the number and type of partners, the value of the relationships and the power dynamics within the relationship play in condom use[19]. To what extent these factors influence condom use in the community may require further study. Awareness raising, behavioral change and condom promotion may be important to develop favorable attitudes towards condom use as a way to prevent the unintended outcomes of unprotected sex.

### Condom use and Associated Factors

Multivariate analysis for condom use during the most recent sex indicated that watching pornography before sex and sex for transactions (money or kind gift) were predictors. Those who watched pornography before sex were less likely to use condom in the most recent sex than those who did not watch i.e. the odds of using condom was 0.39 among those who watched pornography (p<0.001). Similarly, those who received some kind of transactions [money or kind] during most recent sex were less likely to use condom than those who did not receive any gift i.e. the odds of using condom among those who received gifts was 0.39 compared to those who did notreceive gift (p<0.05).

Condom use was largely affected by watching pornography and getting money or material rewards from male sexual partners in the study area. Pornographic media appears to be highly accessible to the youth in the area that might promote unsafe sexual behaviors, which may necessitate establishing strong control and regulatory mechanisms. In kind rewards and transactions for sex, in the prevailing economic problems and impoverished living conditions may reduce negotiation power to use condom among the female youth. Hence, economic empowerment through engagements on small enterprises and other income generating activities that address the reasons for them to engage in such practices may help to reduce risky sexual behaviors.

## Conclusions and Implications

Being a cross-sectional study, it was difficult to establish temporal relationships between variables in the study. Acknowledging this methodological weakness, after adjusting for confounding variables, the study showed predictors of risky sexual behaviors among female youths in Tiss Abay.

The study showed that there was a high prevalence of risky sexual behaviors among female youth in Tiss Abay. Many had multiple and concurrent as well as regular and causal partners. Educating female youth about risky sexual behaviors and ways to cope with the pressures from peers and economic problems should be integral to the existing STIs/HIV and unwanted pregnancy prevention interventions. The 15–19 years age group was found to be less likely to engage on risky sexual behaviors, which can be an entry point for HIV/STIs and unwanted pregnancy prevention education.

Condom use was very low and/or inconsistent, indicating that condom promotion interventions should be targeted to alleviate the prevailing misconceptions and other related dynamics related to not using condom. However, this needs to be supported by making condoms freely available and accessible in the area. The level of substance use was also alarming and associated with risky sexual behavior. As a result, integrating interventions to discourage substance use into promoting healthy sexual behavior interventions may be important.

Pornography appears to be easily accessible and watched by youth, and was associated with not using condoms. Strong controls and regulating pornographic material may be necessary, although difficult with widespread access to the internet.

One third of recent sex acts were conducted against the will of female partners, which violates their rights as human beings, and there is strong demand to educate and mobilize the men and women of the community in this regard.

Finally, further studies in similar settings on the relationship between factors such as unfavorable attitudes and condom use is recommended to ensure that interventions designed to address low proportion of condom use deal with the underlying problems and issues faced by women in such communities.
